# Restoration of Mitochondrial Function Is Essential in the Endothelium-Dependent Vasodilation Induced by Acacetin in Hypertensive Rats

**DOI:** 10.3390/ijms231911350

**Published:** 2022-09-26

**Authors:** Yuan Li, Qingya Dang, Zhiyi Li, Chuting Han, Yan Yang, Miaoling Li, Pengyun Li

**Affiliations:** Key Laboratory of Medical Electrophysiology of Ministry of Education, Medical Electrophysiological Key Laboratory of Sichuan Province, Collaborative Innovation Center for Prevention and Treatment of Cardiovascular Disease, Institute of Cardiovascular Research, Southwest Medical University, Luzhou 646000, China

**Keywords:** acacetin, mitochondria, apoptosis, endothelial dysfunction, hypertension

## Abstract

Mitochondrial dysfunction in the endothelium contributes to the progression of hypertension and plays an obligatory role in modulating vascular tone. Acacetin is a natural flavonoid compound that has been shown to possess multiple beneficial effects, including vasodilatation. However, whether acacetin could improve endothelial function in hypertension by protecting against mitochondria-dependent apoptosis remains to be determined. The mean arterial pressure (MAP) in Wistar Kyoto (WKY) rats, spontaneously hypertensive rats (SHR) administered with acacetin intraperitoneally for 2 h or intragastrically for six weeks were examined. The endothelial injury was evaluated by immunofluorescent staining and a transmission electron microscope (TEM). Vascular tension measurement was performed to assess the protective effect of acacetin on mesenteric arteries. Endothelial injury in the pathogenesis of SHR was modeled in HUVECs treated with Angiotensin II (Ang II). Mitochondria-dependent apoptosis, the opening of Mitochondrial Permeability Transition Pore (mPTP) and mitochondrial dynamics proteins were determined by fluorescence activated cell sorting (FACS), immunofluorescence staining and western blot. Acacetin administered intraperitoneally greatly reduced MAP in SHR by mediating a more pronounced endothelium-dependent dilatation in mesenteric arteries, and the vascular dilatation was reduced remarkably by NG-nitro-L-arginine methyl ester (L-NAME), an inhibitor of NO synthesis. While acacetin administered intragastrically for six weeks had no apparent effect on MAP, it improved the endothelium-dependent dilatation in SHR by activating the AKT/eNOS pathway and protecting against the abnormalities of endothelium and mitochondria. Furthermore, acacetin remarkably inhibited Ang II induced apoptosis by inhibiting the increased expression of Cyclophilin D (CypD), promoted the opening of mPTP, ROS generation, ATP loss and disturbance of dynamin-related protein 1 (DRP1)/optic atrophy1 (OPA1) dynamics in HUVECs. This study suggests that acacetin protected against endothelial dysfunction in hypertension by activating the AKT/eNOS pathway and modulating mitochondrial function by targeting mPTP and DRP1/OPA1-dependent dynamics.

## 1. Introduction

Hypertension is associated with the disruption of vascular homeostasis, leading to the development of endothelial dysfunction and remodeling. Oxidative stress has long been suggested to be one of the driving forces of disease and aging, and mitochondria are both the major source and targets of reactive oxygen species (ROS) [1]. Recent work has emphasized the importance of mitochondria for endothelial function. In contrast to cardiac myocytes and other cell types, even though the endothelium cells (ECs) have low mitochondria content, energy requirements in the endothelium are relatively low, and glycolysis is the major source of ATP production. It is now recognized that the cellular distribution of mitochondria is important for its function and its communication with other cellular organelle (especially endoplasmic reticulum, ER) and nucleus, and endothelial mitochondria play a prominent role in signaling cellular responses to environmental stimuli. Defects in mitochondrial biogenesis and dynamics have detrimental consequences on bioenergetic supply and contribute to the endothelial dysfunction and the pathogenesis of cardiovascular diseases [2,3].

Maintenance of functional mitochondria requires the balance of mitochondrial dynamics, including fission and fusion. Mitochondrial fission is mediated by DRP1, while fusion is executed mostly by OPA1. Defects in mitochondrial biogenesis and the disturbance of OPA1 and DRP1 turnover will result in cell apoptosis and contribute to the endothelial dysfunction and the pathogenesis of cardiovascular diseases [4]. Particularly, CypD is an mPTP structural protein located within the mitochondrial matrix and acts as a Ca^2+^ sensitizer for mPTP opening. The excessive opening of mPTP causes mitochondrial membrane potential collapse, and aggravates the accumulation of mitochondrial ROS. Either genetic knockout or the pharmacological inhibition of CypD could ameliorate mitochondrial dysfunction, including excess mPTP opening and stress [5]. Furthermore, it was reported that CypD could promote the phosphorylation of DRP1 and subsequently augmented DRP1 recruitment to mitochondria, thereby triggering excessive mitochondrial fission and contributing to mitochondrial dysfunction [6]. Additionally, excessive ROS production promotes inflammation and decreases nitric oxide (NO) production and bioavailability, leading to endothelial dysfunction and the development of hypertension, atherosclerosis, pulmonary arterial hypertension and cardiac hypertrophy [7,8,9]. Although the details of the mechanisms responsible for endothelial injury in hypertension are not fully understood, it is generally recognized that mitochondrial dysfunction contributes to endothelial impairment and accelerates the development of hypertension. Concomitantly, many lines of evidence have indicated the therapeutic potential of rescuing mitochondrial homeostasis by targeting mitochondrial fusion or fission pathways in cardiovascular diseases.

**Figure 1:**
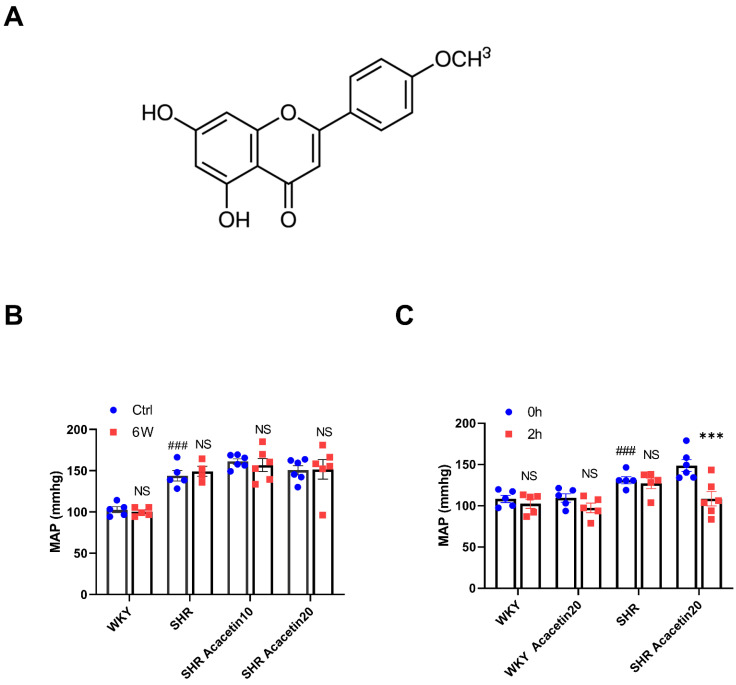
Acacetin reduced MAP in age-matched WKY rats and SHR. (**A**) The molecular structure of acacetin. (**B**) The blood pressure measured in WKY rats and SHR after intragastric administration of acacetin (10 and 20 mg/kg) for six weeks. A statistical analysis is shown by bar graph (NS indicated *p* > 0.05 vs. control group, ### *p* < 0.001 vs. WKY control group, *n* = 5–6 for each group). (**C**) The blood pressure monitored in SHR and WKY rats after being injected intraperitoneally with acacetin at 20 mg/kg. Statistical analysis was shown by bar graph (*** *p* < 0.001 vs. control group (0 h), ### *p* < 0.001 vs. WKY control group (0 h), *n* = 5–6 for each group).

Acacetin (5,7-dihydroxy-4′-methoxyflavone) (Figure 1A) is a natural flavonoid and is present in a variety of plants such as chrysanthemum and safflower. As a traditional Chinese Medicine (TCM), acacetin was suggested to possess numerous pharmacological properties, including anti-cancer, anti-inflammatory, anti-microbial, and anti-proliferative activities, with potential applications in protecting against neuronal dysfunction, cardiac hypertrophy, acute lung injury and atherosclerosis, etc. [10,11,12,13]. In addition, acacetin also showed significant endothelium-dependent vasorelaxant activity [14,15]. Recently, it was reported that acacetin may protect against vascular endothelial cell injury induced by hyperglycemia via preserving mitochondrial function, thereby attenuating diabetes-accelerated atherosclerosis [16]. However, whether acacetin could alleviate the endothelium dysfunction induced by hypertension and the underlying mechanisms remained to be further determined. Therefore, the present study was designed to investigate the protective effect of acacetin against vascular endothelial dysfunction in hypertension.

## 2. Results

### 2.1. Acacetin Administered Intraperitoneally Reduced MAP in SHR

The MAP was measured in WKY rats and age-matched SHR after intragastric administration of acacetin at 10 mg/kg and 20 mg/kg for six weeks. As shown in Figure 1B, the MAP of SHR (137.6 ± 4.22 mmHg) was significantly higher than that of WKY (105.6 ± 2.84 mmHg). There was no significant difference in the MAP measured in SHR+Acacetin group compared with that in SHR after being administered intragastrically with acacetin for six weeks, while intraperitoneal injection of acacetin at 20 mg/kg distinctly decreased the MAP in SHR by 40.20 ± 8.39 mmHg. The MAP in WKY group didn’t show any changes compared with the level before acacetin administration. As the control, the vehicle showed no effect on the MAP in the WKY and SHR group within 2 h (Figure 1C). The above results suggested that the effect of acacetin on blood pressure depended on the route of administration, and intraperitoneal injection of acacetin greatly reduced the MAP in SHR.

### 2.2. Acacetin Improved Endothelium-Dependent Vasodilation in SHR

**Figure 2:**
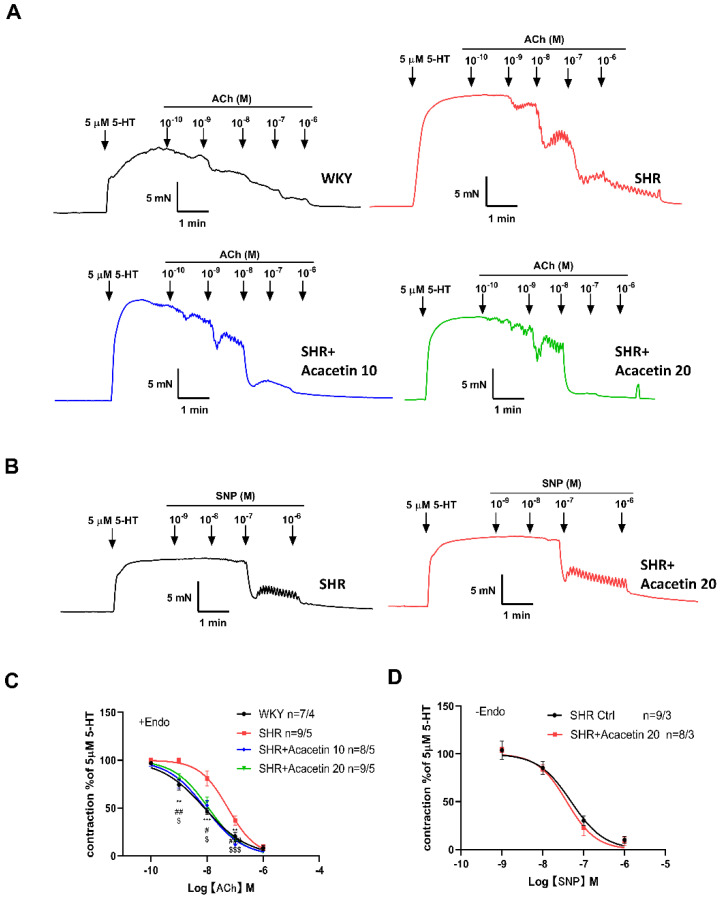
Acacetin improved endothelium-dependent vasodilation in mesenteric arteries of SHR intragastrically administered with acacetin. (**A**) Representative traces showed ACh (1 × 10^−10^ to 1 × 10^−6^ M)–induced vasorelaxation in 5-HT (5 µM)–precontracted endothelium-intact mesenteric arteries from WKY rats, SHR, and SHR administered intragastrically with acacetin at 10 mg/kg (SHR + Acacetin 10) and 20 mg/kg (SHR + Acacetin 20). (**B**) Representative traces showed the vasorelaxant response to SNP in endothelium-denuded (–Endo) mesenteric arteries from SHR and SHR + Acacetin 20. (**C**) Dose-response curves were fitted by a Hill equation (** *p* < 0.01, *** *p* < 0.001 WKY group vs. SHR group; # *p* < 0.05, ## *p* < 0.01, ### *p* < 0.001 SHR + Acacetin 10 group vs. SHR group; $ *p* < 0.05, $$$ *p* < 0.001 SHR + Acacetin 10 group vs. SHR group, *n* = 7–9/4–5, 7–9 indicated the number of arteries; the number of rats was four to five). (**D**) Fitting curves showed the vasorelaxant response to SNP in endothelium-denuded (–Endo) mesenteric arteries from SHR and SHR + Acacetin 20 (*p* > 0.05, *n* = 8–9/3).

In order to check whether the decrease of MAP in SHR was due to the acacetin induced improvement of endothelial function, the relaxation of the mesenteric artery was determined by microvascular ring technique ex vivo. The mesenteric arteries were exposed to 5-hydroxytryptamine (5-HT, 5 μM)–induced vasoconstriction, and relaxations of contracted vessels were assessed by adding cumulative concentrations of ACh (1 × 10^−10^ to 1 × 10^−6^ M). As shown in Figure 2A,C, compared with the WKY rats, ACh–induced vasodilation following 5-HT was apparently attenuated in SHR, while intragastric administration of acacetin at 10 mg/kg or 20 mg/kg (SHR + Acacetin 10, SHR + Acacetin 20) restored endothelial relaxation. Next, after the vascular endothelium was removed (–Endo), acacetin did not change the vasorelaxant response to sodium nitroprusside (SNP) in SHR (Figure 2B,D), suggesting that acacetin had no effect on the vasodilation mediated by smooth muscle cells. Therefore, it was indicated that the intragastric administration of acacetin could improve the endothelium-dependent vasodilation of mesenteric arteries in SHR.

**Figure 3:**
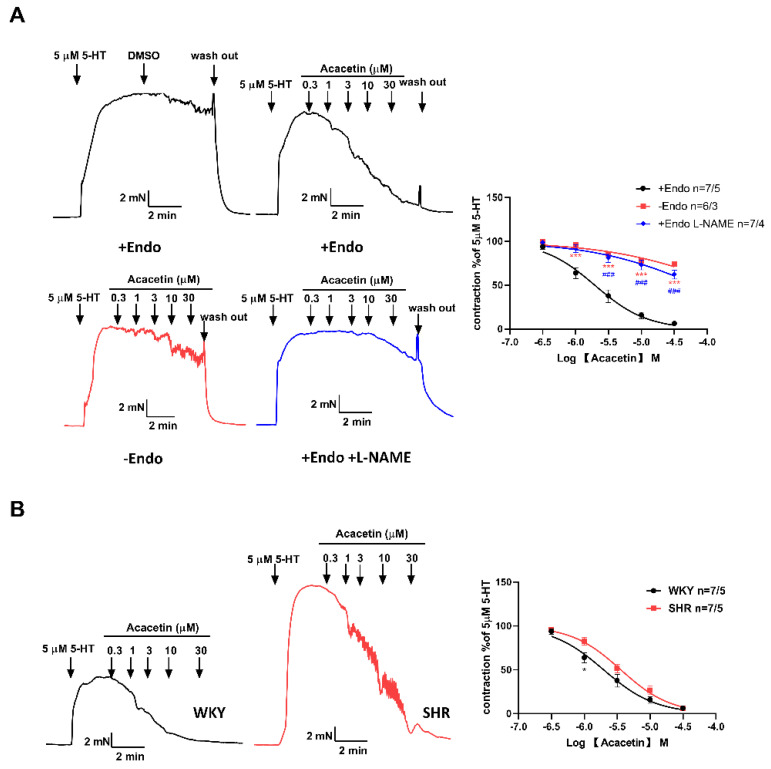
Acacetin mediated endothelium-dependent vasodilation by activation of eNOS. (**A**) Representative traces and fitting curves showed the vasorelaxation responses of acacetin in endothelium-intact (+Endo) and endothelium-denuded (–Endo) arteries. Blocking eNOS activity with L-NAME greatly inhibited acacetin mediated vasodilation (### *p* < 0.001, +Endo L-NAME group vs. +Endo group; *** *p* < 0.001 –Endo group vs. +Endo group; *n* = 6–7/3–5), and DMSO was vehicle control. (**B**) Representative traces and fitting curves showed the vasorelaxation responses of acacetin in endothelium-intact arteries from SHR and WKY rats (* *p* < 0.05, SHR group vs. WKY group, *n* = 7/5).

Concomitantly, the direct effect of acacetin on 5-HT precontracted vessel rings was also determined. Compared with the endothelium-denuded mesenteric arteries, the vasorelaxation response of acacetin in the arteries of WKY rats was more pronounced in endothelium-intact preparations (Figure 3A). In addition, a non-selective NO synthase inhibitor NG-nitro-L-arginine methyl ester (L-NAME, 50 µM) definitively blocked the vasorelaxation response mediated by acacetin in endothelium-intact arteries (Figure 3A), which indicated that acacetin exerted an endothelium-dependent relaxation role through activating nitric oxide synthase (NOS) activity in the endothelium. Furthermore, as shown in Figure 3B, a left shift in the dose-response curve with acacetin exhibiting a greater endothelium-dependent vessel relaxation was detected in WKY rats. Therefore, the above results suggested that acacetin improved vasodilation through activating the NO mediated pathway [17,18].

### 2.3. Acacetin Protected the Endothelium against Mitochondrial Injury in SHR through Activating the AKT/eNOS Pathway

**Figure 4:**
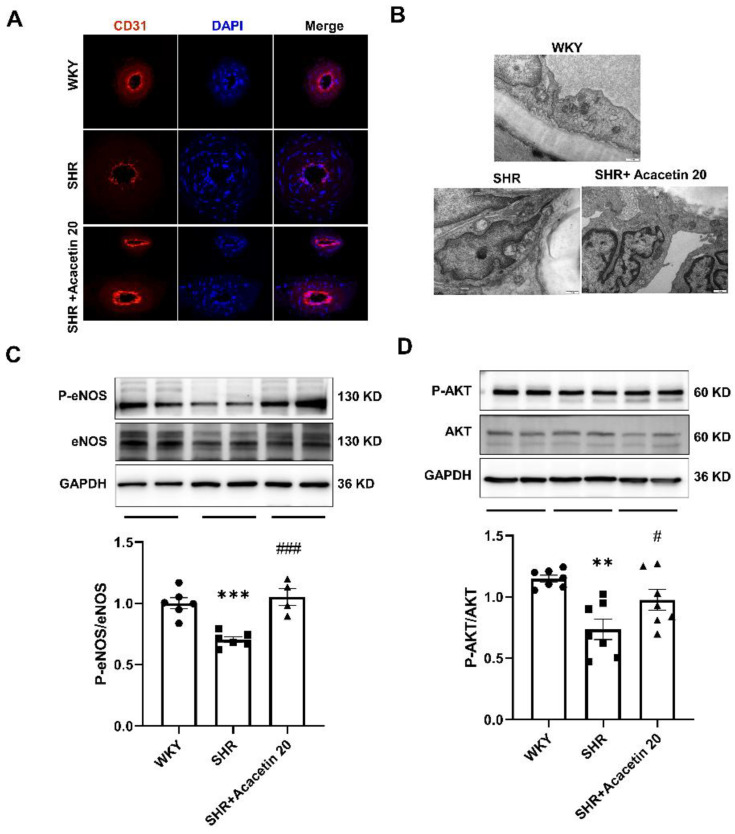
Acacetin reversed endothelial injury in SHR via activating the AKT/eNOS pathway. (**A**) Representative immunofluorescence staining of endothelial injury with specific endothelium marker CD31 (400×). (**B**) Representative transmission electron microscopic images showed the mitochondrial alterations in endothelial cells of mesenteric arteries in different groups (scale bar = 500 nm). (**C**,**D**) Representative western blot bands and quantitative analysis of p-AKT/AKT and p-eNOS/eNOS expressions in mesenteric arteries from WKY, SHR and SHR+Acacetin 20 group (** *p* < 0.01, *** *p* < 0.001 vs. WKY group, # *p* < 0.05, ### *p* < 0.001 vs. SHR group, *n* = 4–7).

To explore the potential mechanisms involved in the endothelium protection mediated by acacetin, a morphological evaluation using immunofluorescent staining and TEM was performed. As shown in Figure 4A, the immunofluorescent staining revealed that the expression of endothelial marker CD31 was reduced, suggesting that the endothelial integrity was damaged in mesenteric arteries from SHR. Accordingly, the electron microscopic images (Figure 4B) showed that the endothelial cells were distinctly attached to the basal lamina, the intercellular junctional complexes were intact, and there were healthy cytoplasmic organelles in the WKY group. However, the endothelial integrity was distorted and abnormal mitochondria with either swollen or irregular cristae were seen in the SHR group, but the administration of acacetin intragastrically for six weeks substantially reversed the abnormal changes of endothelium and mitochondria.

Hypertension linked to defective mitochondrial function are characterized by morphologically abnormal, excessive formation of ROS, uncoupling eNOS and a decrease in the bioavailability of NO, key factors in maintaining the vascular tone [19]. The phosphatidylinositol 3-kinase (PI3K) pathway, which activates serine/threonine protein kinase AKT, enhances eNOS phosphorylation and NO production. Activating the PI3K/AKT/eNOS pathway is central to transducing both mechanical stretch and hormonal inputs to regulate vasodilation [20,21]. Interestingly, increasing studies have revealed that AKT can modulate mitochondria-mediated apoptosis, redox states, dynamic balance, and metabolism. AKT is the core of many signaling pathways and is usually suppressed in many types of mitochondrial dysfunction [22]. As expected, the phosphorylation of eNOS and AKT in mesenteric arteries from the SHR group were greatly reduced by 32.88 ± 7.59% and 29.09 ± 9.05% compared with that in the WKY group, suggesting that long-term treatment with acacetin reversed the reduced protein expression in SHR (Figure 4C,D). Thus, the results suggested that acacetin prevented SHR–induced endothelium dysfunction through activating the AKT/eNOS signal pathway.

### 2.4. Acacetin Suppressed Ang II- Induced Apoptosis in HUVECs

**Figure 5:**
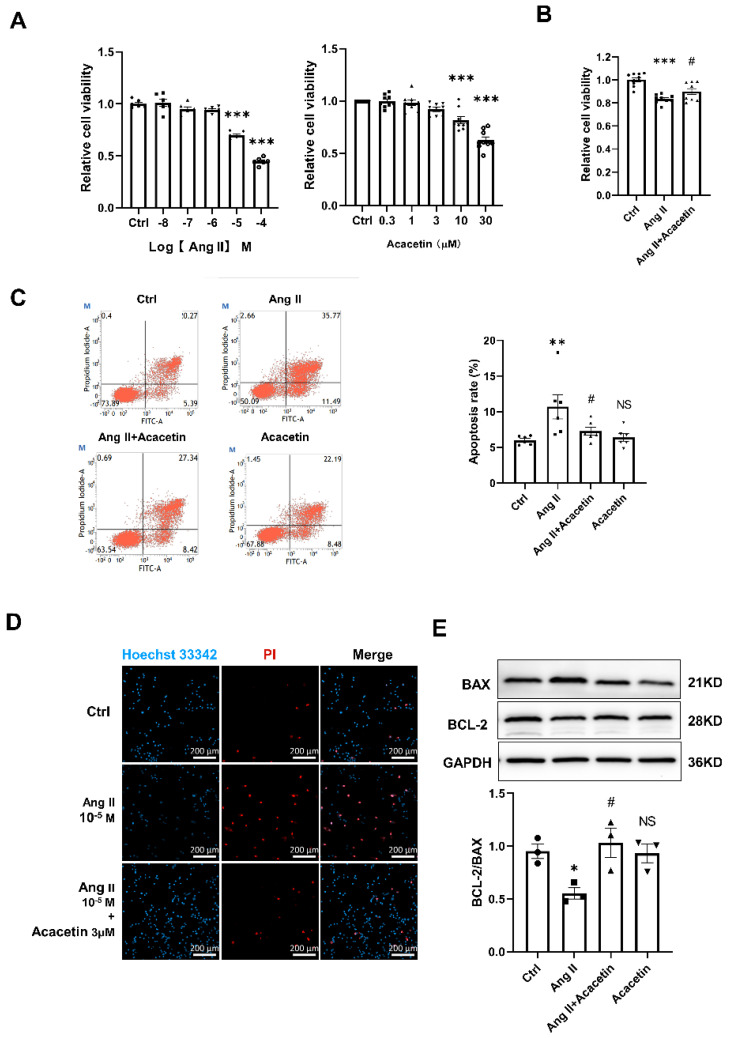
Acacetin inhibited the apoptosis of Ang II–induced HUVECs. (**A**) Cell viability was assayed by CCK8 reagents after incubation with different concentrations of Ang II (1 × 10^−8^ to 1 × 10^−4^ M) or acacetin (0.3 to 30 μM) for 48 h (*n* = 6 for each group). (**B**) Acacetin (3 µM) inhibited the decrease of cell viability induced by Ang II (10 µM) (**C**) Two–parameter dot–plots showed the cell apoptosis determined by annexin V–FITC and PI binding measured by flow cytometry. The percentage of annexin V–positive cells in each treatment was indicated in the bottom right–hand corner of each panel. (**D**) Representative nuclear staining with PI and Hoechst 33342 (Hoechst). Scale bar = 200 μm. (**E**) Western blot analysis showed the protein expression of BCL-2 and BAX in different groups (* *p* < 0.05, ** *p* < 0.01, *** *p* < 0.001 vs. control, # *p* < 0.05 vs. Ang II group. One-way ANOVA with Bonferroni post hoc test was used for group comparisons).

Evidence from previous studies has suggested that dysregulation of the Renin-Angiotensin System during hypertension is associated with increased oxidative stress and endothelial dysfunction [23,24]. In order to clarify the protective mechanisms of acacetin in hypertension, HUVECs were treated by Ang II in vitro. Firstly, a CCK8 assay was performed to assess the effects of Ang II or acacetin alone on HUVECs viability. After treatment for 48 h (Figure 5A), Ang II (1 ×10^−8^ to 1 × 10^−4^ M) dose-dependently inhibited the growth of HUVECs, and particularly at concentrations higher than 1 µM. Meanwhile, acacetin applied at lower concentrations (0.3 to 3 µM) had no effect on the viability of HUVECs but inhibited the cell viability at a concentration of 10 µM and above. Furthermore, pretreatment with acacetin (3 µM) in HUVECs apparently inhibited the Ang II (10 µM)-induced decline of cell viability (Figure 5B). Thus, Ang II (10 µM) and acacetin (3 µM) were chosen as optimum in the following studies.

To further explore whether apoptosis contributed to the suppressed cell viability, FACS was performed in Annexin V–FITC and PI loaded cells pretreated with Ang II or Ang II + Acacetin for 48 h. Compared with the cells in the control or acacetin group, the apoptosis rate of HUVECs was greatly enhanced by Ang II, and reduced by the acacetin intervention (Figure 5C). Consistent with this, Hoechst 33342 and PI double nucleus staining also confirmed the results (Figure 5D). Accordingly, western blot analysis further revealed that, simultaneous to the down regulation of anti-apoptotic protein BCL-2, the expression of pro-apoptotic protein BAX was increased in HUVECs upon treatment with Ang II, and pretreatment with acacetin greatly reversed the expression of BCL-2 and BAX induced by Ang II (Figure 5E). These results suggested that acacetin alleviated the endothelial dysfunction, probably through an anti-apoptotic effect.

### 2.5. Acacetin Protected against Mitochondria-Dependent Apoptosis Induced by Ang II via Regulating the Opening of mPTP and Mitochondrial Dynamics

**Figure 6:**
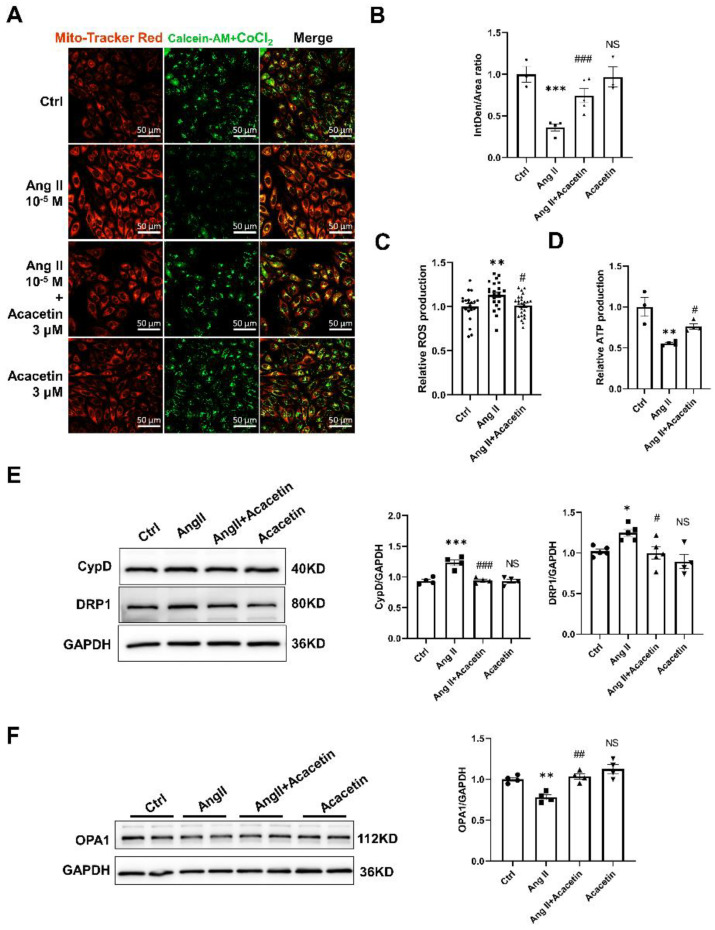
Acacetin inhibited the Ang II mediated mitochondria-dependent apoptosis in HUVECs. (**A**) Representative confocal microscopic images showed that Mitotracker Red confirmed the localization of calcein fluorescence in mitochondria. Calcein/CoCl_2_ was used to detect the opening of mPTP in different groups (the green fluorescence intensity of calcein decreased when the opening of mPTP was prolonged). (**B**) The bar graph showed the quantification of calcein fluorescence in different groups in the presence of CoCl_2_ (*** *p* < 0.001 vs. Control group; ### *p* < 0.001 vs. Ang II group; NS indicated *p* > 0.05 vs. Control group, *n* = 3–5). (**C**) Intracellular ROS was measured by loading the cells with H2DCFDA dye after treatment with compounds as indicated (** *p* < 0.01 vs. Control group; # *p* < 0.05 vs. Ang II group, *n* = 20–25). (**D**) ATP was measured by a CellTiter–Glo^®^ luminescent assay, an indicator of metabolically active cells with normal mitochondria function (** *p* < 0.05 vs. Control group; # *p* < 0.05 vs. Ang II group, *n* = 3). (**E**) Western blot analysis showed the protein expression of CypD and DRP1 in Control, Ang II, Ang II + acacetin and acacetin treated cells. Data were expressed as fold-increase CypD or DRP1 expression normalized to GAPDH. Representative immunoblots were shown on the left (* *p* < 0.05 *** *p* < 0.001 vs. Control group; # *p* < 0.05 ### *p* < 0.001 vs. Ang II group; NS indicated *p* > 0.05 vs. Control group, *n* = 4–5). (**F**) Representative immunoblots and western blot analysis showed the protein expression of OPA1 in control, Ang II treated and Ang II + acacetin treated cells. Data are expressed as fold-increase OPA1 expression normalized to GAPDH (** *p* < 0.01 vs. Control group; ## *p* < 0.01 vs. Ang II group; NS indicated *p* > 0.05 vs. control group, *n* = 4).

To investigate the underlying mechanisms of acacetin in protection against mitochondria-dependent apoptosis in HUVECs elicited by Ang II, we studied the variations in mitochondrial function after Ang II treatment in HUVECs. It was found that the certain indices of mitochondrial function such as mPTP opening indicated by the mean fluorescence intensity of calcein increased by 63.82 ± 12.7% (Figure 6A,B), the ROS level was upregulated by 13.43 ± 4.47% (Figure 6C), and ATP levels decreased by 44.61 ± 8.26% (Figure 6D), while the above indices were greatly inhibited in the cells pretreated with acacetin. These results suggested that acacetin could prevent Ang II triggered mitochondrial dysfunction in HUVECs.

Additionally, accumulative studies have demonstrated that CypD is a regulator of the mPTP, and sensitizes mPTP to calcium and oxidative stress. It can also directly bind to the ATP synthase and reduce ATP production [25]. Consistent with this, the protein expression of CypD in HUVECs treated with Ang II (10 µM) was downregulated, while incubation with acacetin prevented the decrease of CypD (Figure 6E). Recently, it was suggested that CypD could also regulate mitochondrial dynamics in oxidative stress treated cells [26,27]. Furthermore, the OPA1 and DRP1 are crucial for mitochondrial dynamic homeostasis, and the disorder of fusion and fission causes the collapse of the mitochondrial network and promotes apoptosis [28,29]. Similarly, it was confirmed that the expression of OPA1 was greatly downregulated, while DRP1 was obviously upregulated in HUVECs treated with Ang II, while acacetin prevented the above alterations (Figure 6E,F). These results further confirmed that the protective effect of acacetin on the vascular endothelium is due to the preservation of mitochondrial function.

## 3. Discussion

The endothelium regulates vascular tone by influencing the contractile activity of vascular smooth muscle. It has been demonstrated that endothelium-mediated vasodilation is impaired in patients with essential hypertension [1,30]. Novel therapeutic strategies that focused on endothelial relaxation hold great potential for the prevention and treatment of hypertension.

In this study, we found that long-term administration (six weeks) of acacetin effectively improved the mitochondrial function and endothelium-dependent dilation in mesenteric arteries of SHR. Accordingly, a dynamic blood pressure monitor showed that acacetin administered intraperitoneally distinctly decreased the MAP in SHR rather than WKY rats, but the intragastric administration of acacetin didn’t affect the MAP in SHR. This finding is different from previous reports [15], in which it was indicated that acacetin (25 or 50 mg/kg) administrated intragastrically decreased the systolic blood pressure (SBP) in seven-week-old SHR. The inconsistence in findings might be attributed to the different dosage of acacetin and the age of the SHR, thereby influencing the beneficial effect of acacetin. Compared with intragastric administration, intraperitoneal routes are supposed to influence the mesentery, the most vascularized area of the peritoneal cavity, and the compound absorption occurs rather rapidly, but compounds are partially subjected to hepatic first-pass elimination [31,32]. However, whether acacetin could directly influence the excreted vasoactive factors or mediate the interaction between the endothelium and other regulatory molecules remained to be further clarified.

Next, blood pressure mediation is the product of a balance of the cardiac output and the peripheral vascular resistance. Accumulating studies [12,33,34] have demonstrated that long term administration of acacetin could protect against heart dysfunction triggered by ischemia/reperfusion injury, myocardial infarction, cardiomyopathy, etc. Hypertension is characterized with increased myocardial oxygen consumption, impaired epicardial coronary perfusion, ventricular fibrosis, and remodeling [35]. Consistent with these observations, our previous studies have confirmed that acacetin could improve the ejection fraction in SHR, which could be a compensatory mechanism that contributed to the effect of acacetin on MAP in SHR. In addition, it has been demonstrated that the smooth muscle cells in mesenteric arteries directly drive the contraction of the vascular wall and hence regulate the peripheral vascular resistance, which is responsible for the formation of blood pressure [36]. Compared with SHR, the intragastric administration of acacetin (20 mg/kg) had no effect on endothelium-independent dilation induced by SNP (a direct dilator of smooth muscle). Furthermore, no significant differences in the response of vascular tone to vasoconstrictor PE or 5-HT were found between SHR and SHR treated with acacetin (Figure S1 Supplementary Materials), suggesting that the long-term application of acacetin may not affect the vasomotion mediated by smooth muscle in SHR.

The vascular endothelium functions as a dynamic organ which is essential in the regulation of vascular homeostasis. It synthesizes and secretes a series of vasoactive molecules such as NO (vasodilation) or endothelin (vasoconstrictor), responding to physical and chemical signals [32]. The disruption of endothelial function contributes to the progression of hypertension or atherosclerosis, and plays an obligatory role in modulating vascular tone, permeability, and angiogenesis [37,38]. In agreement with these studies, it was found in this study that ACh–induced vasorelaxation was greatly decreased in SHR compared with that in the WKY group. In addition, a specific NO antagonist L-NAME remarkably inhibited the acacetin–mediated vasodilation, suggesting that the vasodilation mediated by acacetin is endothelium-dependent, which is consistent with previous reports [15,17]. Furthermore, acacetin mediated endothelium-dependent dilation in SHR is less significant compared with that in the WKY group, which is consistent with the endothelial injury in SHR. In addition, the vascular tone measurement suggested that long-term treatment of acacetin improved the repaired endothelium-dependent relaxation in SHR, and the changes in endothelium morphology indicated by immunofluorescence and TEM further confirmed this observation. And western blot results also revealed that the long-term administration of acacetin could alleviate the phosphorylation of eNOS and AKT downregulated in mesenteric arteries of SHR. Thus, the above results agree with the potent relaxant effects of acacetin [39], which induce their effect by an activating eNOS in SHR. Similar to our findings, it was reported that acacetin exerted the cardioprotective effects by activating PI3K/AKT signaling [11]. Collectively, our results suggested that an endothelial protective effect of acacetin in SHR is associated with AKT/eNOS activation.

Endothelial dysfunction is not a specific feature of hypertension, but it is common to the majority of cardiovascular risk factors [1]. In particular, patients with hypertension presented greater apoptosis in the endothelium [34,40]. In line with recent reports, cell apoptosis investigated by Annexin V–FITC/PI flow cytometry assay and Hoechst 33342/PI double staining revealed that the percentage of apoptotic cells induced by Ang II was notably reduced by acacetin. Furthermore, western blot analysis confirmed that the expression levels of apoptotic protein BAX was suppressed, while the anti-apoptotic protein BCL-2 was promoted in HUVECs preincubated with acacetin. The above results consistently demonstrated that acacetin could effectively inhibit Ang II induced apoptosis.

Mitochondria are vital mediators of many apoptotic programs whereby the excessive production of ROS, the decreased generation of ATP, the increased mitochondrial permeability, and disordered mitochondrial dynamics ultimately caused apoptosis or necrosis [41]. In particular, the prolonged opening of mPTP is detrimental for mitochondrial function. The calcein/CoCl_2_ technique is commonly used to validate the opening of mPTP [42], and CoCl_2_ could quench the cytosolic signal without affecting the mitochondrial fluorescence in physiological condition. While mitochondrial injury or mPTP inducers could increase the permeability of mitochondria, CoCl_2_ enter the mitochondrial matrix and cause a rapid decrease in mitochondrial calcein fluorescence [6]. Additionally, the increased expression of CypD is associated with the prolonged opening of mPTP [43]. In agreement with others’ reports, our data demonstrated that Ang II elevated the expression level of CypD and enhanced the opening of mPTP, which was indicated by the decrease of the mean fluorescence density of calcein in mitochondria, leading to the disorder of ATP synthesis, increased ROS and accelerated apoptosis. While acacetin could prevent against the upregulation of CypD induced by Ang II in HUVECs, thereby inhibiting the opening of mPTP, ROS generation and ATP deficiency to protect mitochondrial function.

In recent years, the emerging evidence indicated that mitochondrial fusion–fission dynamics play a crucial role in many cellular processes such as cell signaling, apoptosis, and phagocytosis [44]. For example, downregulation or mutations in OPA1 [45], and elevation of cytosolic DRP1 [46,47] will result in increased cell sensitivity to apoptosis. OPA1 and DRP1 governs the delicate balance between fusion and fission in the dynamic mitochondrial network to maintain cellular homeostasis, and excessive fission is a signal of increased mitochondrial damage and leads to enhanced mitophagy to clear dysfunctional mitochondria.

Consistent with these studies, we found that Ang II treatment greatly elevated the protein expression of DRP1 and inhibited OPA1 expression in HUVECs, resulting in the disturbance of mitochondrial dynamics and cell apoptosis, and that acacetin could protect against cell apoptosis by reversing the protein expression of DRP1 and OPA1. Similar findings have also been reported that Ang II induced phosphorylation of DRP1 and mitochondrial fission in abdominal aortic VSMCs and adventitial fibroblasts, which can be inhibited with DRP1 silencing [48,49,50]. Another study suggested that Ang II prompted a shift from punctate to tubular/elongated (fusion) mitochondrial shape in steroidogenic tissues [51]. The difference might be tissue-dependent and the underlying mechanisms remain to be clarified. Additionally, it was reported that targeting the expression or activation of DRP1 might alleviate the inflammatory reactions, contributing to the vessels protection in SHR [52,53]. Therefore, in agreement with previous studies, this result suggested that the disturbance of mitochondrial dynamics was thus likely to contribute to endothelial dysfunction and consequently hypertension, and raised the possibility that interventions directed toward restoring normal mitochondrial dynamics might have a therapeutic benefit.

Furthermore, the limitation of our studies is that the activation of DRP1 or OPA1 is also regulated by a variety of posttranslational modifications such as phosphorylation or acetylation, and several phosphor-sites have been identified, among which phosphorylation of DRP1 or OPA1 can either inhibit or activate its enzymatic activity [54,55]. It was suggested recently that ischemia may cause vascular dysfunction through DRP1-mediated mitochondrial fission [56], and lipid overload activated DRP1 through acetylation, inducing the persistent opening of mPTP and apoptosis, and compromised cardiomyocyte contractile function [54]. Whether the activities of these mitochondrial dynamics proteins have changed during hypertension remain obscure and need to be clarified further. Additionally, ATP was measured here by a luciferase assay, which indirectly reflected the mitochondrial function. Therefore, a direct assessment of mitochondrial dysfunction by measuring the oxygen consumption rate (OCR) in a real-time mode is necessary in our future studies [57]. Although acacetin is a safe and promising candidate for the treatment of cardiovascular diseases, the clinical pharmacological information of acacetin, such as absorption, metabolism and toxicity, is not sufficient at present [33,58]. Therefore, all of these need to be further explored and improved upon to confirm the safety and effectiveness of acacetin in the human body. Overall, there is still a great distance to the routine clinical application of acacetin. However, it cannot be denied that acacetin is a promising natural candidate for cardiovascular drugs.

**Figure 7:**
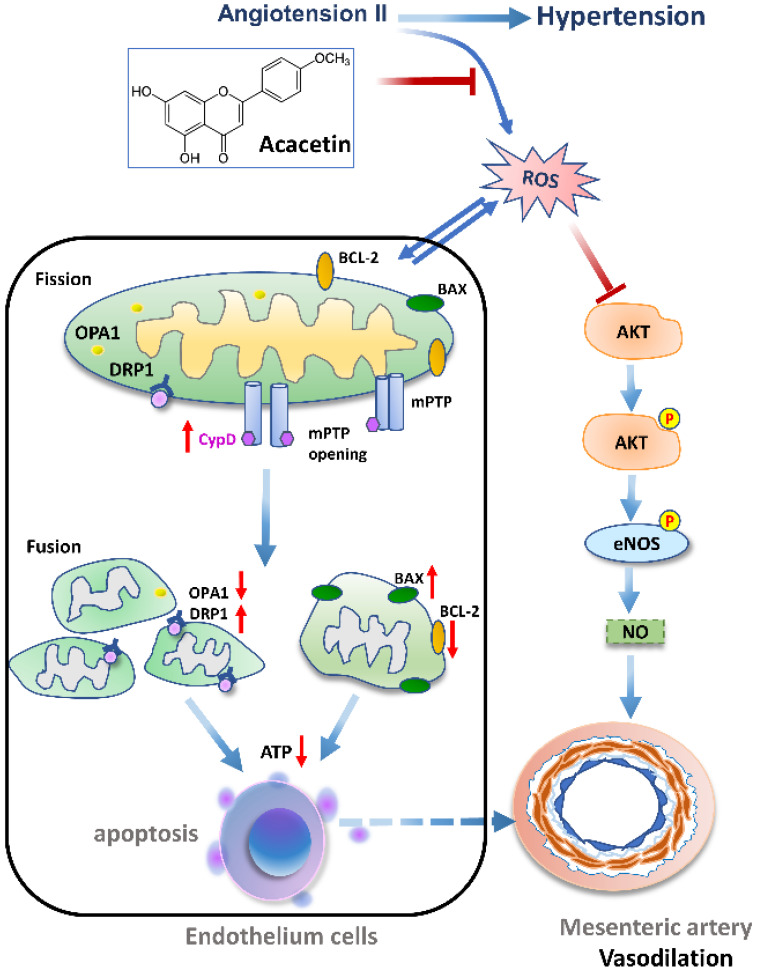
The schematic diagram describing the protective mechanism of acacetin against endothelial injury in hypertension.

In conclusion, our study revealed the links between endothelial dysfunction and mitochondria-dependent apoptosis in mesenteric arteries from SHR (Figure 7). Acacetin apparently normalized the abnormal mitochondrial morphology, protected against oxidative stress induced mitochondrial dysfunction by inhibiting the opening of mPTP, improved mitochondrial dynamics by regulating the balance between DRP1 and OPA1, inhibited excessive ROS generation-induced endothelial cell apoptosis and AKT/eNOS inactivation, and subsequently reversed the decreased endothelium-dependent dilation in hypertension.

## 4. Materials and Methods

### 4.1. Animals

All animal experiments were approved and performed according to the regulations of Southwest Medical University and conform to the guidelines of the Chinese government on the protection of animals used for scientific purposes. The ethical approval code number is SWMU2020664. SHR and age–matched WKY rats were ordered from Vitalriver Co., Ltd. (Beijing, China). The animal license number is SCXK (Jing) 2021–0006. The rats were maintained under specified pathogen–free conditions in a 12 h light–dark cycle, and water and standard food was available to consume ad libitum.

To observe the long-term effect of acacetin on blood pressure, forty male and female SHR and WKY rats, aged 20 weeks and weighing 300–400 g, were randomly assigned to four groups (*n* = 10 rats per group): WKY, SHR, SHR + Acacetin 10 and SHR + Acacetin 20. For the groups of SHR + Acacetin 10 and SHR + Acacetin 20, acacetin was intragastrically administered at a daily dose of 10 mg/kg and 20 mg/kg (*n* = 10 for each group) for six weeks, and WKY and SHR rats were administered an equal volume of vehicle by oral gavage once per day. The femoral artery was then cannulated using a PE 50 catheter to measure the arterial pressure, as previously reported [59].

Meanwhile, to observe the short-term effect of acacetin on blood pressure within 2 h, thirty male and female WKY and SHR rats were randomly assigned to four groups (*n*= 5–6 for each group): WKY, WKY + Acacetin 20, SHR, SHR + Acacetin 20. For the groups of WKY + Acacetin 20 and SHR + Acacetin 20, the rats were intraperitoneally injected with acacetin at a dose of 20 mg/kg. WKY and SHR rats were intraperitoneally injected with equal volume of vehicle.

### 4.2. Detection of Endothelium-Dependent and Independent Vasorelaxation

The mesenteric bed was exposed in rats after they were anesthetized with isoflurane. The second branches of the arteries were carefully dissected by removing the adipose and connective tissue in ice–cold Tyrode’s solution (mM): 137 NaCl, 5.4 KCl, 10 HEPES, 1.2 MgCl_2_, 2.4 CaCl_2_, 10 D-glucose, pH was adjusted to 7.4. The threaded vessel segments (2–4 mm) were transferred and mounted in a myograph chamber which was maintained at 37 °C and pumped with 95% O_2_. After normalization and 30 min equilibration in Tyrode’s solution, the preparations were stimulated with 80 mM KCl. After wash–out and 30 min recovery, contractile responses were evoked with 5-HT (5 µM) or PE (5 µM). When the contraction had reached a plateau, ACh (1 × 10^−10^ to 1 × 10^−6^ M) or SNP (1 × 10^−9^ to 1 × 10^−6^ M) were cumulatively added to the organ bath to induce endothelium-dependent or -independent relaxation. The contraction response was detected using a myograph chamber containing an isometric Mulvany–Halpern myograph (model 610; Danish Myo Technology) and recorded using a PowerLab 8/SP data acquisition system (AD Instruments) as previously described [60]. Relaxations were expressed as the percentage decrease in 5-HT–induced contraction and expressed as means ± SEM.

### 4.3. Immunofluorescence Staining and Transmission Electron Microscopy

After harvesting, the freshly dissected mesenteric arteries were embedded and frozen in OTC compound and cut into 10 µm sections. The sections were rinsed in PBS and blocked with 5% goat serum in PBS containing 0.2% Triton X–100. Subsequently, the sections were incubated with antibody CD31 (1:100 dilution, Cell Signal Technology, Danvers, MA, USA) at 4 °C overnight, followed by incubation with Alexa Fluor^TM^ 546 donkey anti-goat IgG (H + L) for 1 h, and the samples were imaged using a fluorescence microscope.

For TEM, freshly isolated mesenteric arteries were quickly fixed with 3% glutaraldehyde for 24 h, dehydrated in a graded series of ethanol in an increasing concentration, and dried with CO_2_ after being sputter-coated with gold; specimens were imaged using a TEM (JEM–1400–FLASH, JEOL, Tokyo, Japan).

### 4.4. Cell Culture

HUVECs were purchased from the cell bank of the Chinese Academy of Sciences and cultured in an EGM™–2 Endothelial Cell Growth Medium–2 Bullet Kit (CC–3165; Lonza, Basel, Switzerland) with 5% FBS and growth supplements. Cells were first grown in a T25 flask and then seeded on µ–slide 8 well glass bottom slides (80827, Ibidi, Gräfelfing, Germany) for imaging the mPTP opening or cell apoptosis. Cells seeded on a six–well plate were used for immunoblotting, and on a 96 well plate were used for the measurement of ATP or ROS.

### 4.5. Immunoblotting

The total proteins were extracted from rat mesenteric arteries or cultured HUVECs using RIPA lysis buffer containing Halt^TM^ protease and phosphatase inhibitors (78442, Thermo Fisher Scientific, Waltham, MA, USA). The protein concentrations were determined using a BCA protein assay (23225, Thermo Fisher Scientific). Equal amounts of protein from each sample were separated by 10 % SDS–PAGE and then transferred to PVDF membranes (IPVH00010, Millipore, Burlington, MA, USA). Following blocking with 5% BSA in TBS with Tween–20 for 60 min, the membranes were probed with primary antibodies at 4 °C overnight. After being rinsed by TBST, the membranes were further incubated with peroxidase labeled secondary antibody diluted 1:5000 for 60 min at room temperature. The bands were detected by adding chemiluminescent HRP Substrate (Millipore, USA) and quantified using Image J software. The primary antibodies used were specific for p-eNOS (1:500 dilution; Cell Signaling Technology, Danvers, MA, USA), p-AKT (1:500 dilution; Cell Signaling Technology), AKT (1:500 dilution; Cell Signaling Technology, Danvers, MA, USA), eNOS (1:500 dilution; Cell Signaling Technology), BCL-2 (1:500 dilution; Abcam, Cambridge, UK), BAX (1:500 dilution; Abcam), cyclophilin D (CypD, 1:500 dilution, Abcam), OPA1 (1:1000 dilution, Abcam) and GAPDH (1:5000 dilution, Ray antibody Biotech, Peachtree Corners, GA, USA).

### 4.6. Cell Viability and Apoptosis Assays

Cells incubated in 96–well plates were treated as indicated and cell viability was determined using a cell counting kit–8 (CCK8) assay (K1018, APExBIO). The absorbance for CCK8 assay at 460 nm was measured using a spectrophotometer, which is directly proportional to the number of living cells. Apoptosis was measured by an Annexin V–FITC/PI Apoptosis Detection Kit (Procell Life Science & Technology, Hyderabad, India). Briefly, the trypsinized cells were centrifuged and resuspended with a 300 μL 1× binding buffer. Then, 100 μL of cell suspension was transferred into a test tube, and 5 μL of Annexin V–FITC/PI were added successively to each tube and incubated for 15 min at room temperature in the dark. After the addition of 400 μL of 1× binding buffer to each tube, cell apoptosis was analyzed by flow cytometry. Concomitantly, cell apoptosis was also confirmed by Hoechst 33342 and PI double staining. HUVEC monolayers were seeded on glass coverslips and incubated with Hoechst 33342 (1 μM) and PI (15 μM) for 30 min in the dark. The cells were washed with PBS twice and photographed using a fluorescent microscopy.

### 4.7. Measurement of Intracellular ROS, ATP and mPTP Opening

HUVECs were seeded into a 96–well plate at a confluence of 4000 cells per well and then subjected to the different treatments as indicated for 48 h. The cells were washed twice with warm PBS and loaded with H2DCFDA dye at a final concentration of 2 μM in standard bath solution (mM): 130 NaCl, 5 KCl, 8 D–glucose, 10 HEPES, 1.2 MgCl_2_, and 1.5 CaCl_2_, pH 7.4 with NaOH. After incubation for 30 min at 37 °C in the dye, cells were washed with standard bath solution and the fluorescence intensity was read by a microplate reader at an excitation/emission wavelength of 492/520 nm.

Intracellular ATP levels were assayed using a CellTiter–Glo^®^ Luminescent Cell Viability Assay (G7570, Promega, Madison, WI, USA) following the manufacturer’s instructions. Briefly, cells were seeded in a white opaque–bottom 96–well plate, and then subjected to the different treatments for 48 h. CellTiter–Glo Reagent (CellTiter–Glo Buffer + CellTiter–Glo Substrate) was added into the medium and mixed for 2 min on an orbital shaker to induce cell lysis. The luminescence signal was monitored in a Cytation 3 reader (Biotek Instruments, Winooski, VT, USA).

Cells after treatment were coloaded with MitoTracker™ Red FM (M22425, Thermo Fisher) and calcein-AM/CoCl_2_ according to the instructions of the MitoProbe™ Transition Pore Assay Kit (M34253, Thermo Fisher), CoCl_2_ was used for the quenching of the cytosolic signal without affecting the mitochondrial fluorescence.

### 4.8. Chemicals and Drugs

5-hydroxytryptamine (5-HT), phenylephrine (PE), sodium nitroprusside (SNP), and acetylcholine (ACh) were purchased from SigmaAldrich Co. (St. Louis, MO, USA). Acacetin (purity ≧98%) was ordered from Amazigh Pharma Limited (Nanjing, China). All other reagents were analytical grade from local sources. For ex vivo experiments, acacetin was dissolved in DMSO (1% *v*/*v*) and other reagents dissolved in distilled water and sonicated just before use. All other reagents were analytical grade from local sources.

### 4.9. Statistical Analysis

Data were expressed as means ± standard error of the mean (SEM). Statistical analysis was conducted using a paired *t*–test or one–way ANOVA followed by Tukey’s post hoc test. The *p* < 0.05 was considered to be significant. The dose–response curves were plotted and the obtained experimental data were adjusted by a nonlinear curve fitting program (Graphpad prism 9.0).

## Data Availability

The data presented in this study are available upon request from the corresponding author.

## Supplementary Materials

The supporting information can be downloaded at: https://www.mdpi.com/article/10.3390/ijms231911350/s1.
