# Supplementary material for: Restoration of Mitochondrial Function Is Essential in the Endothelium-Dependent Vasodilation Induced by Acacetin in Hypertensive Rats
Source: Int J Mol Sci. 2022 Sep 26;23(19):11350. doi: 10.3390/ijms231911350 (PMC9569784; doi:10.3390/ijms231911350)
Supplement: Supplementary file 1 [file ijms-23-11350-s001.zip › ijms-1917364-supplementary.pdf]

Supplementary Materials:

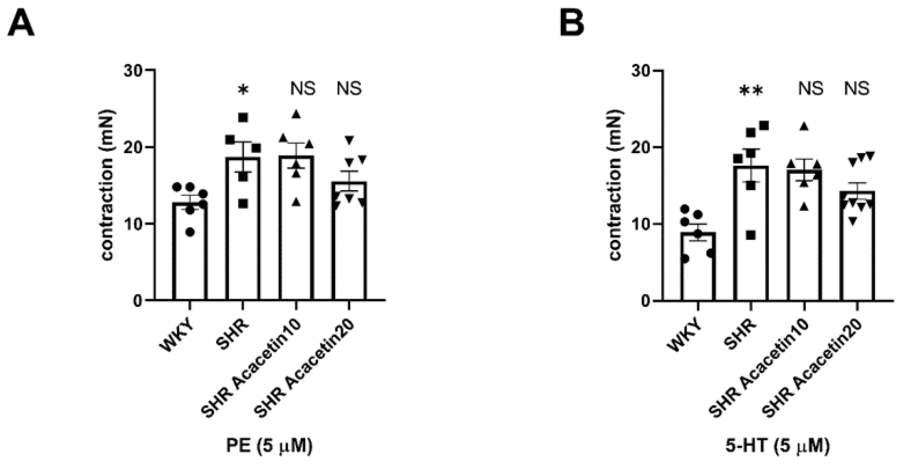

Figure S1. Intragastric administration of acacetin for six weeks had no apparent effect on the vasoconstriction of mesenteric arteries in SHR. A. The maximal contractile force induced by PE (5 μM) in four groups. B. The maximal contractile force induced by 5-HT (5 μM) (*$p < 0.05$, **$p < 0.01$ *vs.* WKY group; NS indicated $p > 0.05$ *vs.* SHR group; n=5-9 for each group).
